# A cyclic carbo-isosteric penta-depsipeptide: *cyclo*(Phe^1^–d-Ala^2^–Gly^3^–Phe^4^–APO^5^)

**DOI:** 10.1107/S2056989014027406

**Published:** 2015-01-01

**Authors:** Stéphanie M. Guéret, Trixie Wagner

**Affiliations:** a Global Discovery Chemistry, Novartis Institutes for Biomedical Research, Novartis International AG, CH-4002 Basel, Switzerland

**Keywords:** crystal structure, depsipeptide, peptidomimetic, carbo-isoster, β-turn, γ-turn

## Abstract

The title compound, *cyclo*(Phe^1^–d-Ala^2^–Gly^3^–Phe^4^–APO^5^), C_26_H_32_N_4_O_5_, is the minor diastereoisomer of a cyclic penta-peptidomimetic analogue containing a novel 2-amino­propyl lactone (APO) motif, which displays the same number of atoms as the native amino acid glycine and has a methyl group in place of the carbonyl O atom. The crystal structure presented here allows the analysis of the secondary structure of this unprecedented cyclic carbo-isosteric depsipeptide. The conformation of the central ring is stabilized by an intra­molecular N—H⋯O hydrogen bond between the carbonyl O atom of the first residue (Phe^1^) and the amide group H atom of the fourth residue (Phe^4^). Based on the previously reported hydrogen bond and on the values of the torsion angles ϕ and ψ, the loop formed by the first, second, third and fourth residues (Phe^1^, d-Ala^2^, Gly^3^ and Phe^4^) can be classified as a type II′ β-turn. The loop around the new peptidomimetic motif, on the other hand, resembles an open γ-turn containing a weak N—H⋯O hydrogen bond between the carbonyl group O atom of the fourth residue (Phe^4^) and the amide unit H atom of the first residue (Phe^1^). In the crystal, the peptidomimetic mol­ecules are arranged in chains along the *b-*axis direction. Within such a chain, the mol­ecules of the structure are linked *via* N—H⋯O hydrogen bonds between the amide group H atom of the secondary residue (d-Ala^2^) and the carb­oxy unit O atom of the fourth residue (Phe^4^) in a neighboring mol­ecule. The newly formed methyl stereocentre of the APO peptidomimetic motif (APO^5^) was obtained as the minor diastereoisomer in a ring-closing reductive amination reaction and adopts an *R* configuration.

## Abstract 2

*Cyclo*(Phe^1^–d-Ala^2^–Gly^3^–Phe^4^–APO^5^) is the minor diastereoisomer of a cyclic penta-peptidomimetic analogue containing a novel 2-amino­propyl lactone (APO) motif, which displays the same number of atoms as the native amino acid glycine and has a methyl group in place of the carbonyl O atom.

## Chemical context   

Cyclic peptidomimetics, with their ability to mimic the secondary structure of peptides, represent a very attractive class of macrocycles. While still being modular and promising a strong affinity for a broad range of biological targets, they have improved pharmacological properties and bioavailability compared to linear peptides. 
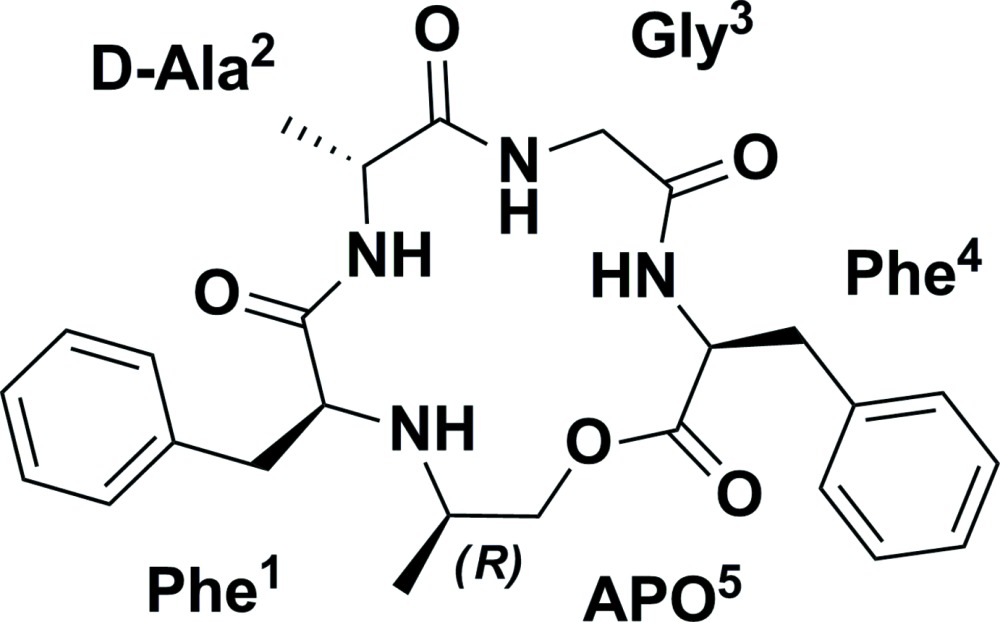


During our research, we have developed a highly selective cyclization method to access a new class of cyclic carbo-isosteric depsipeptides (Guéret *et al.*, 2014 ▸). Our strategy allowed the formation of a novel APO motif which is believed to mimic the glycine amino-acid structure. In order to study the secondary structure of our peptidomimetic motifs, we have started crystallization trials for various analogues. The first compound for which we obtained crystals suitable for single crystal structure determination was the title compound *cyclo*(Phe^1^–d-Ala^2^–Gly^3^–Phe^4^–APO^5^).

## Structural commentary   

**Figure 1:**
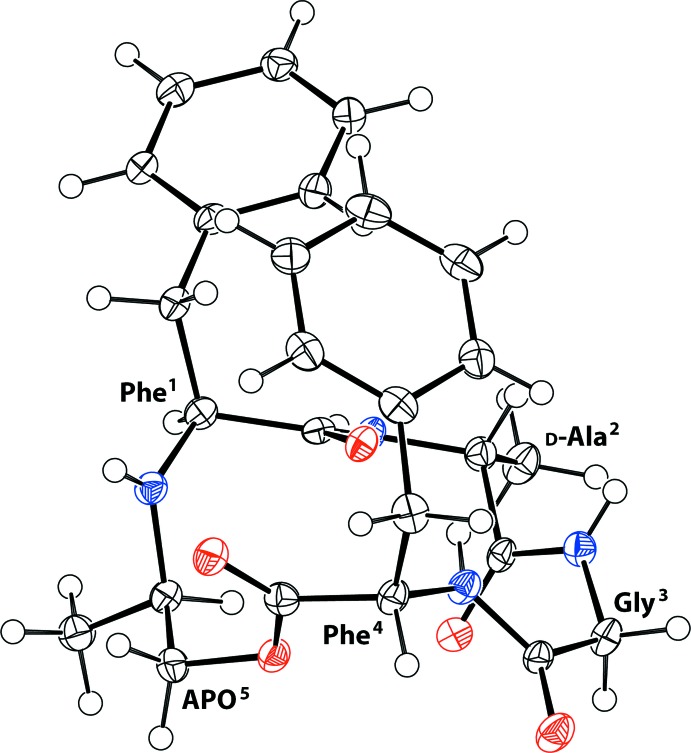
The structure of the title compound in the crystal, including the residue-labelling scheme. Non-H atoms are represented by displacement ellipsoids drawn at the 50% probability level. H atoms are represented as small spheres of arbitrary radius. The atom labelling has been omitted for clarity but is displayed in Fig. 2 ▸.

The cyclic carbo-isosteric depsipeptide *cyclo*(Phe^1^–d-Ala^2^–Gly^3^–Phe^4^–APO^5^) was obtained as the minor diastereoisomer in a ring-closing reductive amination reaction between the C-terminal methyl ketone and the N-terminal amine of phenyl­alanine 1 of the linear precursor H_2_N–Phe^1^–d-Ala^2^–Gly^3^–Phe^4^–CO_2_CH_2_COCH_3_. The two natural amino acids, Phe^1^ and Phe^4^ are in an l-configuration, whereas the unnatural alanine unit, Ala^2^ is in a d-configuration, following the *Cahn–Ingold–Prelog* priority rules or CORN rules (Cahn *et al.*, 1966 ▸). Based on the known stereochemistry of the backbone amino acids, the absolute configuration of the newly formed methyl stereocentre α to the secondary amine (N9) of the minor diastereoisomer could be unambiguously assigned as C19*R.* The result is supported by a Flack *x* parameter of 0.10 (11), calculated using the quotient method (Parsons & Flack, 2004 ▸) as implemented in the 2013 version of *SHELXL* (Sheldrick, 2008 ▸). The structure of the title compound in the crystal, including the residue-labelling scheme, is shown in Fig. 1 ▸.

**Figure 2:**
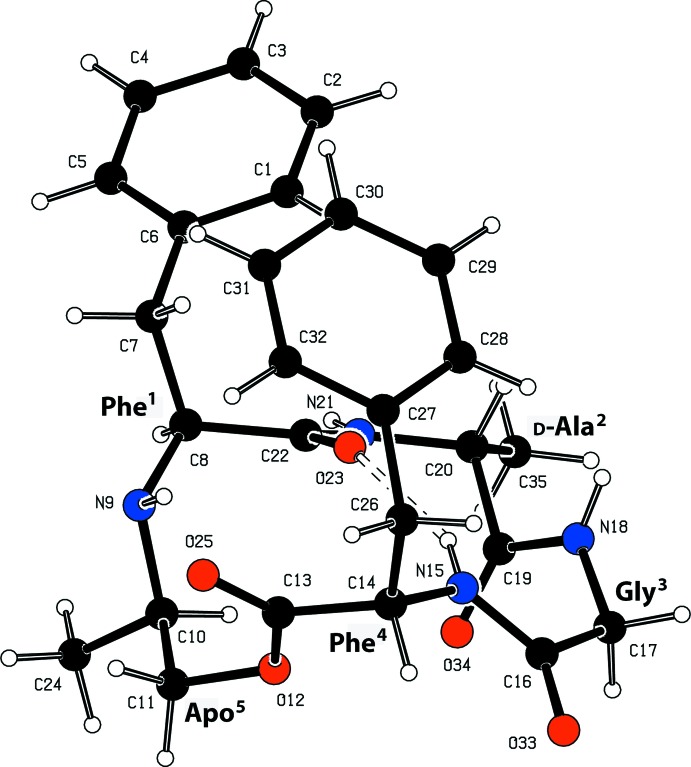
The atom- and residue-labelling scheme of the title compound, showing the intra­molecular hydrogen bond. All atoms are represented as small spheres of arbitrary radius.

The secondary structure of the cyclic peptidomimetic, in which all peptidic bonds adopt a *trans* conformation, is stabilized by a β-turn containing an intra­molecular hydrogen bond (Table 1 ▸, Fig. 2 ▸) between the carbonyl oxygen O23 of the first residue (Phe^1^) and the amide hydrogen N15—H15 of the residue located three residues after the first residue (Phe^4^). The related torsion angle values fall into the corresponding type II′ β-turn Ramachandran plot area (Ramachandran *et al.*, 1963 ▸). The APO peptidomimetic motif adopts an open γ-turn with a loose hydrogen bond between the carbonyl oxygen of the lactone unit (O25) of the first residue (Phe^4^) and the secondary amine (N9) of the residue located two residues after the first (Phe^1^). Selected backbone torsion angles are given in Table 2 ▸ and a review on the secondary structure of peptides and proteins is given by Smith *et al*. (1980 ▸).

## Supra­molecular features   

**Figure 3:**
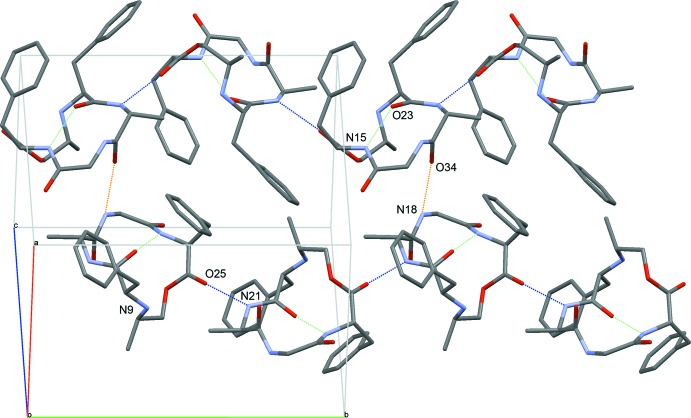
Packing diagram along the face diagonal of the plane defined by the *a* and *c* axes, showing the hydrogen-bonded chains parallel to *b*. Hydrogen bonds are indicated as green (intra­molecular), blue (inter­molecular within the chains) and orange (inter­molecular between chains) dotted lines. H atoms have been omitted for clarity.

The *cyclo*(Phe^1^–d-Ala^2^–Gly^3^–Phe^4^–APO^5^) mol­ecules align in the crystal in infinite chains parallel to the *b* axis (Fig. 3 ▸). Within each chain, the peptide mol­ecules are linked *via* hydrogen bonds between O25 and N21—H21 (blue). The individual chains are loosely connected via hydrogen bonds between O34 and N18—H18 (orange).

## Synthesis and crystallization   

**Step 1** The linear precursor H_2_N-Phe^1^–d-Ala^2^–Gly^3^–Phe^4^–CO_2_CH_2_COCH_3_ (90.7 mg, 152 µmol) was stirred in hydrogen chloride (4 *M* in 1,4-dioxane, 20.0 ml) at 0° C for 1 h, then at room temperature for 2 h. The reaction mixture was concentrated under reduced pressure and the resulting amine was used in the following step without further purification. **Step 2** The previously obtained crude amine was dissolved in DMF (15.2 ml) and acetic acid (152 µl, 2.66 mmol) was added. The reaction mixture was stirred at room temperature for 1.5 h. **Step 3** To the imine reaction mixture, sodium cyano­borohydride (11.5 mg, 182 µmol) was added followed by methanol (3.80 ml), leading to a final concentration of 8 m*M* with a 1:4 ratio of MeOH/DMF. The resulting reaction mixture was stirred at room temperature for 16 h and then concentrated under vacuum. The crude residue was directly purified by preparative RP-HPLC on an Atlantis Prep T3 OBD (30 × 150 mm; 5 µm) column at a flow rate of 60 ml/min with a step gradient of 5 to 15% for 2.5 min, 15 to 35% for 12 min, 35 to 45% for 2 min, then 45 to 95% for 0.1 min of MeCN in H_2_O + 0.1% TFA. Selected fractions were combined and lyophilized to yield the desired cyclic peptidomimetic (65.0 mg, 85%) as a white fluffy solid, TFA salt and a 81:19 mixture of two diastereoisomers. A fraction of the purified mixture of diastereoisomers (29 mg) was re-purified by preparative chiral-HPLC using a Chiralpak (20 × 250 mm; 5 µm) column at a flow rate of 12 ml/min with an optimized *n*-hepta­ne/*i*-PrOH/MeOH/DEA (80:18:2:0.03) isocratic gradient to afford the major diastereoisomer (16.0 mg, d.e. = 98.9%) as a desalted white fluffy solid and the minor diastereoisomer (3.2 mg, d.e. = 99.4%) as desalted white fluffy solid.

**Crystallization of minor diastereoisomer** Crystals of the title compound were obtained by dissolving the minor diastereo­isomer in a minimum amount of ethyl acetate and *n*-heptane (1:1) from which the solvents were allowed to slowly evapor­ate at room temperature.

**Analytical data of the crystalline minor diastereoisomer** HRMS (ESI) calculated for C_26_H_33_N_4_O_5_ [*M* + H]^+^: 481.2541, found 481.2448. IR (neat) ν_max_/cm^−1^ 3335 (*br*), 3065, 3035, 2940, 1730, 1675 (*br*), 1545, 1480, 1455, 1205, 1135, 750, 725, 700. ^1^H NMR (600 MHz, (CD_3_)_2_SO) 8.81 (1H, *dd*, *J* = 7.2 and 5.2 Hz, NH), 8.59 (1H, *d*, *J* = 4.6 Hz, NH), 7.85 (1H, *d*, *J* = 9.7 Hz, NH), 7.33–7.16 (10H, *m*, 2 × Phe-5ArH), 4.77 (1H, *td*, *J* = 9.3 and 6.0 Hz, Phe-Hα), 3.97 (1H, *dd*, *J* = 7.1 and 4.6 Hz, Ala-Hα), 3.86–3.73 (3H, *m*, OC*H*_2_ and Gly-Hα), 3.41 (1H, *t*, *J* = 7.3 Hz, Phe-Hα), 3.36–3.34 (2H, *m*, Gly-Hα), 3.18 (1H, *dd*, *J* = 13.9 and 6.0 Hz, Phe-Hβ), 2.94 (1H, *dd*, *J* = 13.9 and 9.0 Hz, Phe-Hβ), 2.68 (1H, *dd*, *J* = 13.6 and 6.5 Hz, Phe-Hβ), 2.62 (1H, *dd*, *J* = 13.6 and 8.0 Hz, Phe-Hβ), 2.59–2.53 (1H, *m*, C*H*CH_3_), 2.18 (1H, *s*, NHamine), 1.12 (3H, *d*, *J* = 7.0 Hz, Ala-3Hβ), 0.78 (3H, *d*, *J* = 6.4 Hz, CHC*H*_3_). ^13^C NMR (150 MHz, (CD_3_)_2_SO) 176.5, 173.9, 170.2, 168.5, 138.6, 137.3, 129.2 (4 × CH), 128.4 (2 × CH), 127.9 (2 × CH), 126.6, 126.1, 67.5, 60.2, 52.8, 51.3, 50.1, 42.5, 39.5, 37.6, 17.4, 15.9.

## Refinement   

Crystal data, data collection and structure refinement details are summarized in Table 3 ▸. The C-bound H atoms were calculated in idealized positions (C–H = 0.98–1.00 Å) and refined using a riding model with *U*_iso_(H) = 1.2*U*_eq_(parent atom). The hydrogen atoms of the amide groups and the hy­droxy group were located in a difference Fourier map and allowed to refine freely.

## Supplementary Material

Crystal structure: contains datablock(s) I. DOI: 10.1107/S2056989014027406/lh5744sup1.cif

Structure factors: contains datablock(s) I. DOI: 10.1107/S2056989014027406/lh5744Isup2.hkl

Supporting information file. DOI: 10.1107/S2056989014027406/lh5744Isup3.cml

CCDC reference: 1039448

Additional supporting information:  crystallographic information; 3D view; checkCIF report

## Figures and Tables

**Table 1:** Hydrogen-bond geometry (, )

*D*H*A*	*D*H	H*A*	*D* *A*	*D*H*A*
N9H9O25	0.95(3)	2.49(3)	3.338(3)	149(2)
N15H15O23	0.83(3)	2.08(3)	2.853(3)	155(2)
N18H18O34^i^	0.87(3)	2.29(3)	3.163(4)	177(3)
N21H21O25^ii^	0.80(3)	2.18(3)	2.949(3)	161(3)

Symmetry codes: (i) 
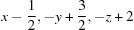
; (ii) 
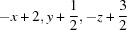
.

**Table 2:** Selected backbone torsion angles ()

Phe^1^	C10N9C8C22	1	61.6(2)
Phe^1^	N9C8C22N21	1	131.8(2)
D-Ala^2^	C22N21C20C19	2	55.4(2)
D-Ala^2^	N21C20C19N18	2	134.2(2)
Gly^3^	C19N18C17C16	3	79.0(3)
Gly^3^	N18C17C16N15	3	4.0(3)
Phe^4^	C16N15C14C13	4	121.6(2)
Phe^4^	N15C14C13O12	4	40.3(2)
APO^5^	C13O12C11C10	5	103.6(2)
APO^5^	O12C11C10N9	5	77.0(2)

**Table 3:** Experimental details

Crystal data	
Chemical formula	C_26_H_32_N_4_O_5_
*M*_r_	480.56
Crystal system, space group	Orthorhombic, *P*2_1_2_1_2_1_
Temperature (K)	100
*a*, *b*, *c* ()	10.126(9), 15.096(14), 15.355(13)
*V* (^3^)	2347(4)
*Z*	4
Radiation type	Cu *K*
(mm^1^)	0.78
Crystal size (mm)	0.12 0.07 0.05

Data collection	
Diffractometer	Bruker *SMART* 6000 CCD
Absorption correction	Multi-scan (*SADABS*; Sheldrick, 1999 ▸)
*T*_min_, *T*_max_	0.486, 0.753
No. of measured, independent and observed [*I* > 2(*I*)] reflections	23011, 4133, 3847
*R*_int_	0.075
(sin /)_max_ (^1^)	0.595

Refinement	
*R*[*F* ^2^ > 2(*F* ^2^)], *wR*(*F* ^2^), *S*	0.047, 0.112, 1.09
No. of reflections	4133
No. of parameters	330
H-atom treatment	H atoms treated by a mixture of independent and constrained refinement
_max_, _min_ (e ^3^)	0.31, 0.43
Absolute structure	Flack *x* determined using 1590 quotients [(*I* ^+^)(*I* )]/[(*I* ^+^)+(*I* )] (Parsons Flack, 2004 ▸)
Absolute structure parameter	0.10(11)

Computer programs: *SMART* (Bruker, 2003 ▸), *SAINT* (Bruker, 2004 ▸), *SHELXS97* and *SHELXL97* (Sheldrick, 2008 ▸), *PLATON* (Spek, 2009 ▸) and *publCIF* (Westrip, 2010 ▸).

## supplementary crystallographic information

### Crystal data

**Table:**

C_26_H_32_N_4_O_5_	*D*_x_ = 1.360 Mg m^−^^3^
*M**_r_* = 480.56	Cu *K*α radiation, λ = 1.54178 Å
Orthorhombic, *P*2_1_2_1_2_1_	Cell parameters from 9940 reflections
*a* = 10.126 (9) Å	θ = 4.1–68.8°
*b* = 15.096 (14) Å	µ = 0.78 mm^−^^1^
*c* = 15.355 (13) Å	*T* = 100 K
*V* = 2347 (4) Å^3^	Block, colourless
*Z* = 4	0.12 × 0.07 × 0.05 mm
*F*(000) = 1024	

### Data collection

**Table:**

Bruker SMART 6000 CCD diffractometer	4133 independent reflections
Radiation source: Microstar rotating anode generator	3847 reflections with *I* > 2σ(*I*)
Incoatec multilayer mirrors monochromator	*R*_int_ = 0.075
Detector resolution: 5.6 pixels mm^-1^	θ_max_ = 66.6°, θ_min_ = 4.1°
ω scans	*h* = −12→12
Absorption correction: multi-scan (*SADABS*; Sheldrick, 1999)	*k* = −17→17
*T*_min_ = 0.486, *T*_max_ = 0.753	*l* = −18→18
23011 measured reflections	

### Refinement

**Table:**

Refinement on *F*^2^	Secondary atom site location: difference Fourier map
Least-squares matrix: full	Hydrogen site location: inferred from neighbouring sites
*R*[*F*^2^ > 2σ(*F*^2^)] = 0.047	H atoms treated by a mixture of independent and constrained refinement
*wR*(*F*^2^) = 0.112	*w* = 1/[σ^2^(*F*_o_^2^) + (0.0764*P*)^2^] where *P* = (*F*_o_^2^ + 2*F*_c_^2^)/3
*S* = 1.09	(Δ/σ)_max_ = 0.011
4133 reflections	Δρ_max_ = 0.31 e Å^−^^3^
330 parameters	Δρ_min_ = −0.43 e Å^−^^3^
0 restraints	Absolute structure: Flack *x* determined using 1590 quotients [(*I*^+^)-(*I*^-^)]/[(*I*^+^)+(*I*^-^)] (Parsons & Flack, 2004)
0 constraints	Absolute structure parameter: 0.10 (11)
Primary atom site location: structure-invariant direct methods	

### Special details

**Table:**

Geometry. All e.s.d.'s (except the e.s.d. in the dihedral angle between two l.s. planes) are estimated using the full covariance matrix. The cell e.s.d.'s are taken into account individually in the estimation of e.s.d.'s in distances, angles and torsion angles; correlations between e.s.d.'s in cell parameters are only used when they are defined by crystal symmetry. An approximate (isotropic) treatment of cell e.s.d.'s is used for estimating e.s.d.'s involving l.s. planes.
Refinement. Refinement of *F*^2^ against ALL reflections. The weighted *R*-factor *wR* and goodness of fit *S* are based on *F*^2^, conventional *R*-factors *R* are based on *F*, with *F* set to zero for negative *F*^2^. The threshold expression of *F*^2^ > σ(*F*^2^) is used only for calculating *R*-factors(gt) *etc*. and is not relevant to the choice of reflections for refinement. *R*-factors based on *F*^2^ are statistically about twice as large as those based on *F*, and *R*- factors based on ALL data will be even larger.

### Fractional atomic coordinates and isotropic or equivalent isotropic displacement parameters (Å^2^)

**Table:**

	*x*	*y*	*z*	*U*_iso_*/*U*_eq_	
C1	0.6916 (2)	0.75967 (14)	0.68262 (14)	0.0209 (4)	
H1	0.6894	0.7333	0.7388	0.025*	
C2	0.6031 (2)	0.82691 (14)	0.66282 (14)	0.0227 (4)	
H2	0.5431	0.8475	0.7058	0.027*	
C3	0.6026 (2)	0.86397 (14)	0.58022 (15)	0.0223 (4)	
H3	0.5413	0.9095	0.5663	0.027*	
C4	0.6915 (2)	0.83465 (15)	0.51801 (14)	0.0231 (4)	
H4	0.6904	0.8594	0.4611	0.028*	
C5	0.7827 (2)	0.76883 (14)	0.53901 (13)	0.0206 (4)	
H5	0.8450	0.7501	0.4966	0.025*	
C6	0.7835 (2)	0.73002 (14)	0.62175 (13)	0.0191 (4)	
C7	0.8833 (2)	0.65948 (13)	0.64407 (13)	0.0191 (4)	
H7A	0.9203	0.6354	0.5893	0.023*	
H7B	0.8374	0.6105	0.6744	0.023*	
C8	0.9979 (2)	0.69186 (13)	0.70179 (13)	0.0178 (4)	
H8	1.0399	0.7448	0.6741	0.021*	
N9	1.09594 (18)	0.62140 (11)	0.70982 (11)	0.0188 (4)	
H9	1.056 (2)	0.5716 (19)	0.7369 (18)	0.023*	
C10	1.2144 (2)	0.64565 (14)	0.76043 (13)	0.0196 (4)	
H10	1.1909	0.6941	0.8021	0.024*	
C11	1.2647 (2)	0.56634 (14)	0.81143 (14)	0.0213 (4)	
H11A	1.2632	0.5130	0.7739	0.026*	
H11B	1.3571	0.5770	0.8297	0.026*	
O12	1.18248 (15)	0.55144 (9)	0.88820 (9)	0.0204 (3)	
C13	1.0956 (2)	0.48517 (13)	0.88625 (14)	0.0196 (4)	
C14	1.0182 (2)	0.48060 (14)	0.97123 (13)	0.0198 (4)	
H14	1.0780	0.4536	1.0158	0.024*	
N15	0.98462 (18)	0.56809 (12)	1.00179 (11)	0.0191 (4)	
H15	0.949 (3)	0.6021 (18)	0.9664 (19)	0.023*	
C16	1.0227 (2)	0.59836 (14)	1.08029 (13)	0.0184 (4)	
C17	0.9816 (2)	0.69272 (14)	1.10269 (13)	0.0212 (4)	
H17A	0.9236	0.6904	1.1545	0.025*	
H17B	1.0619	0.7261	1.1194	0.025*	
N18	0.9139 (2)	0.74228 (11)	1.03547 (11)	0.0197 (4)	
H18	0.828 (3)	0.7378 (18)	1.0336 (17)	0.024*	
C19	0.9831 (2)	0.77969 (13)	0.96966 (13)	0.0175 (4)	
C20	0.8968 (2)	0.82801 (14)	0.90145 (13)	0.0197 (4)	
H20	0.8026	0.8097	0.9090	0.024*	
N21	0.94164 (18)	0.80253 (12)	0.81497 (12)	0.0188 (4)	
H21	0.953 (3)	0.8414 (19)	0.7802 (19)	0.023*	
C22	0.94722 (18)	0.71732 (13)	0.79294 (13)	0.0165 (4)	
O23	0.91694 (15)	0.65798 (9)	0.84460 (9)	0.0196 (3)	
C24	1.3230 (2)	0.67838 (15)	0.69969 (15)	0.0236 (5)	
H24A	1.3504	0.6301	0.6610	0.035*	
H24B	1.3988	0.6982	0.7342	0.035*	
H24C	1.2896	0.7279	0.6647	0.035*	
O25	1.08111 (16)	0.43461 (10)	0.82577 (10)	0.0245 (3)	
C26	0.8961 (2)	0.42006 (14)	0.96385 (15)	0.0238 (5)	
H26A	0.9245	0.3609	0.9434	0.029*	
H26B	0.8563	0.4128	1.0223	0.029*	
C27	0.7928 (2)	0.45570 (14)	0.90239 (15)	0.0222 (5)	
C28	0.7071 (2)	0.52271 (15)	0.92819 (15)	0.0244 (5)	
H28	0.7078	0.5426	0.9869	0.029*	
C29	0.6201 (2)	0.56087 (15)	0.86874 (17)	0.0275 (5)	
H29	0.5631	0.6072	0.8872	0.033*	
C30	0.6156 (2)	0.53217 (15)	0.78319 (16)	0.0271 (5)	
H30	0.5568	0.5590	0.7428	0.033*	
C31	0.6983 (2)	0.46364 (15)	0.75708 (15)	0.0254 (5)	
H31	0.6950	0.4426	0.6988	0.030*	
C32	0.7853 (2)	0.42612 (15)	0.81573 (15)	0.0245 (5)	
H32	0.8413	0.3793	0.7971	0.029*	
O33	1.08418 (17)	0.55439 (10)	1.13399 (10)	0.0268 (4)	
O34	1.10326 (14)	0.77972 (10)	0.96627 (9)	0.0220 (3)	
C35	0.9080 (3)	0.92754 (14)	0.91424 (15)	0.0258 (5)	
H35A	1.0002	0.9458	0.9067	0.039*	
H35B	0.8783	0.9431	0.9730	0.039*	
H35C	0.8527	0.9579	0.8712	0.039*	

### Atomic displacement parameters (Å^2^)

**Table:**

	*U*^11^	*U*^22^	*U*^33^	*U*^12^	*U*^13^	*U*^23^
C1	0.0215 (10)	0.0231 (10)	0.0180 (10)	−0.0008 (9)	0.0012 (8)	0.0021 (8)
C2	0.0219 (10)	0.0258 (10)	0.0206 (11)	0.0004 (9)	0.0018 (8)	−0.0027 (8)
C3	0.0211 (10)	0.0214 (10)	0.0244 (11)	0.0019 (9)	−0.0031 (9)	−0.0020 (8)
C4	0.0258 (11)	0.0238 (10)	0.0197 (10)	−0.0018 (9)	−0.0035 (9)	0.0019 (9)
C5	0.0214 (11)	0.0239 (11)	0.0163 (10)	0.0001 (8)	−0.0017 (8)	−0.0022 (8)
C6	0.0191 (10)	0.0199 (10)	0.0183 (10)	−0.0038 (8)	−0.0030 (8)	−0.0015 (8)
C7	0.0221 (10)	0.0192 (10)	0.0160 (9)	−0.0009 (8)	−0.0005 (8)	−0.0017 (8)
C8	0.0202 (10)	0.0172 (9)	0.0161 (9)	−0.0002 (8)	−0.0009 (8)	0.0029 (7)
N9	0.0199 (9)	0.0186 (9)	0.0180 (9)	0.0007 (7)	−0.0003 (7)	−0.0003 (7)
C10	0.0206 (11)	0.0209 (10)	0.0174 (10)	−0.0004 (8)	−0.0006 (8)	−0.0028 (8)
C11	0.0191 (10)	0.0262 (11)	0.0186 (10)	0.0001 (8)	0.0023 (8)	0.0007 (9)
O12	0.0213 (7)	0.0245 (8)	0.0153 (7)	−0.0021 (6)	0.0003 (6)	0.0002 (6)
C13	0.0207 (10)	0.0161 (9)	0.0221 (10)	0.0023 (8)	−0.0013 (8)	0.0006 (8)
C14	0.0248 (11)	0.0190 (10)	0.0157 (9)	0.0008 (9)	−0.0005 (8)	0.0021 (8)
N15	0.0253 (9)	0.0165 (8)	0.0155 (9)	0.0030 (7)	−0.0023 (7)	0.0015 (7)
C16	0.0187 (10)	0.0229 (10)	0.0137 (9)	−0.0011 (8)	0.0009 (8)	0.0029 (8)
C17	0.0284 (11)	0.0204 (10)	0.0148 (10)	−0.0001 (9)	0.0004 (9)	−0.0001 (7)
N18	0.0205 (9)	0.0203 (9)	0.0183 (9)	0.0002 (7)	0.0008 (7)	0.0006 (7)
C19	0.0228 (11)	0.0151 (9)	0.0145 (9)	0.0016 (8)	0.0000 (8)	−0.0030 (7)
C20	0.0217 (10)	0.0193 (10)	0.0182 (10)	0.0019 (9)	0.0007 (8)	−0.0003 (8)
N21	0.0260 (9)	0.0169 (9)	0.0134 (8)	−0.0004 (7)	0.0001 (7)	0.0023 (7)
C22	0.0154 (9)	0.0188 (10)	0.0153 (10)	−0.0001 (7)	−0.0038 (7)	0.0012 (8)
O23	0.0240 (7)	0.0194 (7)	0.0153 (7)	−0.0010 (6)	0.0002 (6)	0.0011 (5)
C24	0.0233 (10)	0.0239 (10)	0.0237 (11)	0.0002 (9)	−0.0006 (9)	−0.0003 (8)
O25	0.0272 (8)	0.0244 (7)	0.0219 (8)	0.0005 (7)	−0.0011 (6)	−0.0057 (6)
C26	0.0273 (11)	0.0189 (10)	0.0251 (11)	−0.0004 (9)	0.0024 (9)	0.0027 (8)
C27	0.0200 (10)	0.0173 (10)	0.0294 (11)	−0.0052 (8)	0.0027 (9)	0.0024 (8)
C28	0.0225 (11)	0.0234 (10)	0.0273 (11)	−0.0047 (9)	0.0041 (9)	−0.0040 (9)
C29	0.0189 (11)	0.0236 (11)	0.0399 (14)	−0.0003 (9)	0.0004 (9)	−0.0027 (10)
C30	0.0207 (10)	0.0233 (11)	0.0374 (13)	−0.0024 (9)	−0.0028 (9)	0.0022 (10)
C31	0.0220 (11)	0.0266 (11)	0.0275 (12)	−0.0054 (9)	−0.0004 (9)	−0.0038 (9)
C32	0.0220 (11)	0.0205 (10)	0.0309 (11)	−0.0016 (9)	0.0025 (9)	−0.0034 (9)
O33	0.0357 (9)	0.0265 (8)	0.0183 (7)	0.0055 (7)	−0.0044 (7)	0.0016 (6)
O34	0.0221 (8)	0.0232 (7)	0.0206 (7)	−0.0003 (6)	−0.0016 (6)	−0.0003 (6)
C35	0.0357 (12)	0.0195 (10)	0.0221 (10)	0.0034 (10)	−0.0016 (9)	−0.0014 (8)

### Geometric parameters (Å, º)

**Table:**

C1—C2	1.387 (3)	C16—C17	1.523 (3)
C1—C6	1.393 (3)	C17—N18	1.447 (3)
C1—H1	0.9500	C17—H17A	0.9900
C2—C3	1.386 (3)	C17—H17B	0.9900
C2—H2	0.9500	N18—C19	1.353 (3)
C3—C4	1.385 (3)	N18—H18	0.87 (3)
C3—H3	0.9500	C19—O34	1.218 (3)
C4—C5	1.394 (3)	C19—C20	1.546 (3)
C4—H4	0.9500	C20—N21	1.455 (3)
C5—C6	1.399 (3)	C20—C35	1.519 (3)
C5—H5	0.9500	C20—H20	1.0000
C6—C7	1.507 (3)	N21—C22	1.331 (3)
C7—C8	1.540 (3)	N21—H21	0.80 (3)
C7—H7A	0.9900	C22—O23	1.235 (3)
C7—H7B	0.9900	C24—H24A	0.9800
C8—N9	1.460 (3)	C24—H24B	0.9800
C8—C22	1.539 (3)	C24—H24C	0.9800
C8—H8	1.0000	C26—C27	1.508 (3)
N9—C10	1.475 (3)	C26—H26A	0.9900
N9—H9	0.95 (3)	C26—H26B	0.9900
C10—C11	1.519 (3)	C27—C28	1.391 (3)
C10—C24	1.524 (3)	C27—C32	1.406 (4)
C10—H10	1.0000	C28—C29	1.393 (4)
C11—O12	1.461 (3)	C28—H28	0.9500
C11—H11A	0.9900	C29—C30	1.384 (4)
C11—H11B	0.9900	C29—H29	0.9500
O12—C13	1.333 (3)	C30—C31	1.390 (4)
C13—O25	1.211 (3)	C30—H30	0.9500
C13—C14	1.523 (3)	C31—C32	1.381 (4)
C14—N15	1.442 (3)	C31—H31	0.9500
C14—C26	1.542 (3)	C32—H32	0.9500
C14—H14	1.0000	C35—H35A	0.9800
N15—C16	1.345 (3)	C35—H35B	0.9800
N15—H15	0.83 (3)	C35—H35C	0.9800
C16—O33	1.228 (3)		
			
C2—C1—C6	121.3 (2)	N18—C17—C16	116.85 (18)
C2—C1—H1	119.4	N18—C17—H17A	108.1
C6—C1—H1	119.4	C16—C17—H17A	108.1
C3—C2—C1	119.9 (2)	N18—C17—H17B	108.1
C3—C2—H2	120.1	C16—C17—H17B	108.1
C1—C2—H2	120.1	H17A—C17—H17B	107.3
C4—C3—C2	120.0 (2)	C19—N18—C17	120.2 (2)
C4—C3—H3	120.0	C19—N18—H18	121.6 (18)
C2—C3—H3	120.0	C17—N18—H18	117.1 (18)
C3—C4—C5	119.9 (2)	O34—C19—N18	123.31 (19)
C3—C4—H4	120.0	O34—C19—C20	122.35 (19)
C5—C4—H4	120.0	N18—C19—C20	114.24 (19)
C4—C5—C6	120.81 (19)	N21—C20—C35	110.87 (18)
C4—C5—H5	119.6	N21—C20—C19	108.49 (17)
C6—C5—H5	119.6	C35—C20—C19	109.68 (18)
C1—C6—C5	118.1 (2)	N21—C20—H20	109.3
C1—C6—C7	121.49 (19)	C35—C20—H20	109.3
C5—C6—C7	120.40 (18)	C19—C20—H20	109.3
C6—C7—C8	114.32 (17)	C22—N21—C20	120.04 (18)
C6—C7—H7A	108.7	C22—N21—H21	122.1 (19)
C8—C7—H7A	108.7	C20—N21—H21	117.4 (19)
C6—C7—H7B	108.7	O23—C22—N21	121.80 (19)
C8—C7—H7B	108.7	O23—C22—C8	119.05 (18)
H7A—C7—H7B	107.6	N21—C22—C8	119.11 (18)
N9—C8—C22	109.38 (16)	C10—C24—H24A	109.5
N9—C8—C7	109.26 (17)	C10—C24—H24B	109.5
C22—C8—C7	110.56 (17)	H24A—C24—H24B	109.5
N9—C8—H8	109.2	C10—C24—H24C	109.5
C22—C8—H8	109.2	H24A—C24—H24C	109.5
C7—C8—H8	109.2	H24B—C24—H24C	109.5
C8—N9—C10	114.60 (16)	C27—C26—C14	113.00 (18)
C8—N9—H9	109.2 (15)	C27—C26—H26A	109.0
C10—N9—H9	107.9 (15)	C14—C26—H26A	109.0
N9—C10—C11	110.42 (17)	C27—C26—H26B	109.0
N9—C10—C24	110.16 (18)	C14—C26—H26B	109.0
C11—C10—C24	109.20 (18)	H26A—C26—H26B	107.8
N9—C10—H10	109.0	C28—C27—C32	117.8 (2)
C11—C10—H10	109.0	C28—C27—C26	120.9 (2)
C24—C10—H10	109.0	C32—C27—C26	121.1 (2)
O12—C11—C10	110.26 (17)	C27—C28—C29	120.6 (2)
O12—C11—H11A	109.6	C27—C28—H28	119.7
C10—C11—H11A	109.6	C29—C28—H28	119.7
O12—C11—H11B	109.6	C30—C29—C28	120.9 (2)
C10—C11—H11B	109.6	C30—C29—H29	119.6
H11A—C11—H11B	108.1	C28—C29—H29	119.6
C13—O12—C11	118.28 (16)	C29—C30—C31	119.2 (2)
O25—C13—O12	124.8 (2)	C29—C30—H30	120.4
O25—C13—C14	124.47 (19)	C31—C30—H30	120.4
O12—C13—C14	110.74 (17)	C32—C31—C30	120.1 (2)
N15—C14—C13	111.01 (17)	C32—C31—H31	120.0
N15—C14—C26	112.16 (19)	C30—C31—H31	120.0
C13—C14—C26	112.10 (18)	C31—C32—C27	121.4 (2)
N15—C14—H14	107.1	C31—C32—H32	119.3
C13—C14—H14	107.1	C27—C32—H32	119.3
C26—C14—H14	107.1	C20—C35—H35A	109.5
C16—N15—C14	122.33 (19)	C20—C35—H35B	109.5
C16—N15—H15	120.0 (18)	H35A—C35—H35B	109.5
C14—N15—H15	117.2 (18)	C20—C35—H35C	109.5
O33—C16—N15	124.3 (2)	H35A—C35—H35C	109.5
O33—C16—C17	119.49 (19)	H35B—C35—H35C	109.5
N15—C16—C17	116.21 (18)		
			
C6—C1—C2—C3	2.0 (3)	O33—C16—C17—N18	177.65 (19)
C1—C2—C3—C4	−0.9 (3)	N15—C16—C17—N18	−4.0 (3)
C2—C3—C4—C5	−1.0 (3)	C16—C17—N18—C19	−79.0 (3)
C3—C4—C5—C6	1.7 (3)	C17—N18—C19—O34	−5.5 (3)
C2—C1—C6—C5	−1.2 (3)	C17—N18—C19—C20	178.25 (17)
C2—C1—C6—C7	177.58 (19)	O34—C19—C20—N21	49.5 (3)
C4—C5—C6—C1	−0.6 (3)	N18—C19—C20—N21	−134.16 (19)
C4—C5—C6—C7	−179.45 (19)	O34—C19—C20—C35	−71.7 (3)
C1—C6—C7—C8	−75.1 (3)	N18—C19—C20—C35	104.6 (2)
C5—C6—C7—C8	103.7 (2)	C35—C20—N21—C22	175.89 (19)
C6—C7—C8—N9	−172.90 (16)	C19—C20—N21—C22	55.4 (2)
C6—C7—C8—C22	66.7 (2)	C20—N21—C22—O23	−1.1 (3)
C22—C8—N9—C10	−61.6 (2)	C20—N21—C22—C8	−178.64 (17)
C7—C8—N9—C10	177.27 (17)	N9—C8—C22—O23	−45.8 (2)
C8—N9—C10—C11	145.33 (18)	C7—C8—C22—O23	74.5 (2)
C8—N9—C10—C24	−94.0 (2)	N9—C8—C22—N21	131.84 (19)
N9—C10—C11—O12	−77.0 (2)	C7—C8—C22—N21	−107.8 (2)
C24—C10—C11—O12	161.69 (17)	N15—C14—C26—C27	59.4 (2)
C10—C11—O12—C13	103.6 (2)	C13—C14—C26—C27	−66.3 (2)
C11—O12—C13—O25	1.6 (3)	C14—C26—C27—C28	−78.9 (3)
C11—O12—C13—C14	−179.37 (16)	C14—C26—C27—C32	97.2 (2)
O25—C13—C14—N15	−140.7 (2)	C32—C27—C28—C29	−2.1 (3)
O12—C13—C14—N15	40.3 (2)	C26—C27—C28—C29	174.1 (2)
O25—C13—C14—C26	−14.4 (3)	C27—C28—C29—C30	1.0 (3)
O12—C13—C14—C26	166.61 (17)	C28—C29—C30—C31	0.7 (3)
C13—C14—N15—C16	−121.6 (2)	C29—C30—C31—C32	−1.2 (3)
C26—C14—N15—C16	112.1 (2)	C30—C31—C32—C27	0.0 (3)
C14—N15—C16—O33	−2.8 (3)	C28—C27—C32—C31	1.7 (3)
C14—N15—C16—C17	178.95 (19)	C26—C27—C32—C31	−174.6 (2)

### Hydrogen-bond geometry (Å, º)

**Table:**

*D*—H···*A*	*D*—H	H···*A*	*D*···*A*	*D*—H···*A*
N9—H9···O25	0.95 (3)	2.49 (3)	3.338 (3)	149 (2)
N15—H15···O23	0.83 (3)	2.08 (3)	2.853 (3)	155 (2)
N18—H18···O34^i^	0.87 (3)	2.29 (3)	3.163 (4)	177 (3)
N21—H21···O25^ii^	0.80 (3)	2.18 (3)	2.949 (3)	161 (3)

Symmetry codes: (i) *x*−1/2, −*y*+3/2, −*z*+2; (ii) −*x*+2, *y*+1/2, −*z*+3/2.

## References

[bb1] Bruker (2003). *SMART*. Bruker AXS Inc., Madison, Wisconsin, USA.

[bb2] Bruker (2004). *SAINT*. Bruker AXS Inc., Madison, Wisconsin, USA.

[bb3] Cahn, R. S., Ingold, C. K. & Prelog, V. (1966). *Angew. Chem. Int. Ed. Engl.* **5**, 385–415.

[bb4] Guéret, S. M., Meier, P. & Roth, H. J. (2014). *Org. Lett.* **16**, 1502–1505. 10.1021/ol5003797 24571727

[bb5] Parsons, S. & Flack, H. (2004). *Acta Cryst.* A**60**, s61.

[bb6] Ramachandran, G. N., Ramakrishnan, C. & Sasisekharan, V. (1963). *J. Mol. Biol.* **7**, 95–99. 10.1016/s0022-2836(63)80023-6 13990617

[bb7] Sheldrick, G. M. (1999). *SADABS*. University of Göttingen, Germany.

[bb8] Sheldrick, G. M. (2008). *Acta Cryst.* A**64**, 112–122. 10.1107/S0108767307043930 18156677

[bb9] Smith, J. A., Pease, L. G. & Kopple, K. D. (1980). *Crit. Rev. Biochem. Mol. Biol.* **8**, 315–399. 10.3109/10409238009105470 7002463

[bb10] Spek, A. L. (2009). *Acta Cryst.* D**65**, 148–155. 10.1107/S090744490804362X PMC2631630 19171970

[bb11] Westrip, S. P. (2010). *J. Appl. Cryst.* **43**, 920–925.

